# Striking a balance: how long physical activity is ideal for academic success? Based on cognitive and physical fitness mediation analysis

**DOI:** 10.3389/fpsyg.2023.1226007

**Published:** 2023-07-13

**Authors:** Guoqing Liu, Wenjie Li, Xiaotian Li

**Affiliations:** ^1^Institute of Physical Education and Training, Capital University of Physical Education and Sports, Beijing, China; ^2^School of Recreation and Community Sport, Capital University of Physical Education and Sports, Beijing, China

**Keywords:** structural equation modeling, physical activity, academic achievement, cognitive ability, physical fitness, mediation analysis

## Abstract

Balancing physical activity and studying is an important issue facing Chinese teenagers. Therefore, numerous studies have found that engaging in physical activity can promote academic performance among students. However, what is the optimal duration of physical activity? This study used data from the China Education Panel Survey, with a sample size of 18,009 valid respondents. General linear regression analysis was conducted using Stata 17 software to evaluate the effects of different durations of physical activity, cognitive ability, and physical Fitness on academic performance among adolescents. Furthermore, the “Bootstrap Mediation” method and the “Maximum Likelihood Estimation” method were used to analyze whether physical health and cognitive ability have significant mediating effects. The present study reveals the following findings: (1) There exists a non-linear relationship between students’ academic performance and the duration of physical exercise. The greatest improvement in academic performance is observed when the exercise duration reaches 2 hours. (2) Physical exercise can enhance students’ academic performance by promoting physical health and cognitive abilities. (3) Gender heterogeneity is observed, with the optimal exercise duration for male students being 2 hours, while female students exhibit the highest academic performance when exercising for one hour. This study provides theoretical guidance for research on adolescents’ participation in physical Activity and improving academic performance, enabling adolescents to engage in physical Activity more scientifically and rationally.

## 1. Introduction

In 2022, the World Health Organization (WHO) recommended that adolescents engage in at least 60 min of moderate to vigorous physical activity (PA) per day, every day of the week (World Health Organization, 2022). However, according to the “Guidelines on Physical Activity and Sedentary Behavior” released by the WHO, approximately 81% of adolescents worldwide do not meet the recommended level of physical activity (Guthold et al., 2020). In China, only 23.8% of students have achieved the standard for health and fitness (News, 2021). On the one hand, academic performance is undeniably one of the most valued aspects within the educational system, driven by the prevailing exam-oriented ideology. However, physical exercise is often overlooked and placed at the bottom of the educational agenda, resulting in significant academic pressure faced by adolescents (Wunsch et al., 2017), adolescents lack time to exercise. On the other hand, there is a widespread belief in contemporary society that academic achievement and physical exercise are contradictory, leading to social biases against student participation in physical activities (Hao and Yi, 2022), which holds that physical exercise takes up study time and consumes energy that is detrimental to learning. In addition, many parents arrange various types of cultural classes for their children on the weekend, depriving them of time for physical exercise (Pei and Gao, 2013). This misconception and neglect of physical exercise have led to the increasing prominence of sub-health issues among adolescents. However, is this perception accurate? What is the actual relationship between physical exercise and academic achievement? And through what mechanisms does physical exercise influence academic performance? These questions have been the subject of extensive discussions and research in fields such as education and psychology, attracting widespread attention and concern from various sectors of society.

Currently, there is limited research on the correlation between Physical activity duration, cognitive ability, Physical fitness, and academic achievement. However, many studies have explored the relationship between Physical activity and academic achievement. For example, a data analysis of a sample size of 10,205 showed that physical activity can improve classroom behavior and benefit academic achievement (Álvarez-Bueno et al., 2017). A randomized controlled trial from Spain found that moderate to high-intensity physical activity can improve academic achievement (Visier-Alfonso et al., 2021). In a 14-week randomized controlled trial in Australia involving 500 students, adding 1.25 h of endurance training per day did not negatively affect academic achievement (Dwyer et al., 1983), and subsequent follow-up data showed an upward trend in academic achievement (Maynard et al., 1987). Therefore, the viewpoint that Physical activity can improve academic achievement has been widely supported (Bangsbo et al., 2016; Reed, 2016; McPherson et al., 2018). In addition, a longitudinal study with a sample size of 5,316 found that longer Physical activity sessions per week had a greater impact on academic achievement for female students, but had no effect on male students (Carlson et al., 2008). Research has found that academic and cognitive performance are important factors that influence the health of students (Masoomi et al., 2020). An American cross-sectional study conducted over two semesters found no correlation between attending physical education class and academic achievement, but subsequent analyses found that self-initiated intense physical activity outside of class significantly improved students’ academic achievement (Coe et al., 2006). These two studies suggest that there may be heterogeneity between male and female students, and that Physical activity outside of physical education classes can impact academic achievement.

In contrast, a study of 708 Hong Kong teenagers found that 22.54% of participants met the daily 60-min exercise requirement, but their academic achievement did not improve (Chan and Hui, 2016). In another study in China involving 333 Hong Kong students, academic achievement was measured by their exam results, and the results showed that the level of physical activity was unrelated to academic achievement (Yu et al., 2006). Singh’s review of 11 high-quality intervention studies found no correlation between physical activity and academic achievement improvement (Singh et al., 2019). A Canadian cross-sectional study with a sample size of 6,923 found a positive correlation between physical activity and self-esteem, but a small negative correlation with academic achievement (Tremblay et al., 2000). Based on these studies, it can be concluded that there are conflicting views in existing literature on whether Physical activity affects academic achievement, This may be primarily because researchers have overlooked that different durations of Physical activity may have different effects on students’ academic achievement. Physical activity duration may have an optimal value, and when this time is exceeded, Physical activity may have a negative impact on students’ academic achievement. In addition, the arousal theory suggests that appropriate duration of physical exercise can awaken the functional levels of the body and mind in adolescents (Hillman et al., 2008). Therefore, based on the above research and common sense, this article proposes the hypothesis that different durations of Physical activity have different effects on students’ academic achievement.

However, how does Physical activity affect academic achievement? Some studies suggest that Physical activity can improve academic achievement (Reed, 2016; Tomporowski and Pesce, 2019) by improving Physical fitness level and enhancing cognitive ability. Since physical exercise can enhance cognitive abilities and improve academic performance, can it also improve academic performance by enhancing physical fitness levels? Therefore, based on the above findings and hypotheses, this article explores the effects of different durations of Physical activity on students’ academic achievement, examines the mediating effect of cognitive ability and Physical fitness on the relationship between Physical activity and academic achievement. This study provides theoretical guidance for research on adolescent participation in Physical activity and academic achievement, guides adolescents to participate in Physical activity scientifically, and improves society’s understanding of Physical activity, breaking down their misconceptions about how Physical activity affects academic achievement.

## 2. Materials and methods

### 2.1. Data sources

This study utilized data from the China Education Panel Study (CEPS), which was conducted by the National Survey Research Center (NSRC) at Renmin University of China. The baseline survey focused on students in the seventh and ninth grades. The sampling design for CEPS employed a multi-stage probability proportionate to size (PPS) method. The sampling process involved four stages: In the first stage (PSU), 28 counties (districts) were selected from county-level administrative units nationwide. In the second stage (SSU), within each selected county (district), four schools offering seventh and ninth grades were chosen within their respective geographic areas. In the third stage (TSU), four classes were selected from each sampled school, including two classes from the seventh grade and two classes from the ninth grade. Finally, in the fourth stage, all students, parents, homeroom teachers, subject teachers (Chinese, math, and English), and school administrators from the selected classes constituted the final survey sample. The data collection process involved randomly selecting 438 classes from 112 schools located in 28 county-level units (counties, districts, and cities) across China, ensuring good national representativeness. The CEPS dataset not only collected basic information such as students’ height, weight, cognitive ability, and academic achievement but also included rich family and school-related information. A total of 19,487 students were surveyed at baseline, and after excluding samples with missing values, 18,009 valid samples remained (see Table 1).

**Table 1:** Descriptive statistical analysis of variables.

Var Name	Variable definition	Obs	Mean	SD	Min	Max
new_var	Continuous variable, final grade: translated into a standard score with a mean of 70	18,009	210.050	26.505	27.16	293.93
mz	Category variable, ethnicity; 0 = other ethnicity, 1 = Han ethnicity	18,009	1.910	0.280	1	2
jiaoyu	Categorical variable, education level among parents: education level of the highest parent; 1 = none, 2 = elementary school, 3 = junior high school, 4 = high school, 5 = college and above	18,009	3.570	0.906	1	5
pla23	Category variable, type of area where the school is located; 1 = central urban area of the city/county, 2 = fringe urban area of the city/county, 3 = rural/urban area of the city/county, 4 = town outside the city/county area, 5 = rural	18,009	2.640	1.565	1	5
grade9	Category variable, grade level; 0 = 7th grade, 1 = 9th grade	18,009	0.470	0.499	0	1
sthktype	Category variable, student’s current hukou type; 0 = non-agricultural hukou, 1 = agricultural hukou	18,009	0.540	0.498	0	1
nl	Continuous variable, age	18,009	14.510	1.240	12	18
steco_3c	Category variable, student’s family economic status; 1 = difficult, 2 = moderate, 3 = rich	18,009	1.850	0.496	1	3
b01	Whether the child is an only child, 0 = non-only child, 1 = only child	18,009	0.440	0.496	0	1
a01	Gender, 0 = female, 1 = male	18,009	0.510	0.500	0	1
zmdl4	Category variable, weekend exercise time; 0 = no exercise, 1 = 1 h, 2 = 2 h, 3 = 3 h	18,009	0.770	1.006	0	3
bmi2	Physical fitness, 0 = unhealthy, 1 = healthy	18,009	0.630	0.484	0	1
cog3pl	Continuous variable, standardized scores on cognitive ability tests (using 3PL model)	18,009	0.020	0.857	−2.03	2.71

### 2.2. Research methods

This article mainly uses two statistical methods. Firstly, a general linear regression model is used to evaluate the effects of cognitive ability and Physical fitness on academic achievement of adolescents under different durations of Physical activity. The linear regression model is used to analyze whether there are significant differences in the effects of cognitive ability and Physical fitness level on academic achievement of adolescents under different exercise duration conditions. Secondly, the “Bootstrap mediation” method and maximum likelihood estimation (ML) method are used to determine whether Physical fitness and cognitive ability have significant mediating effects. Bootstrap is the most widely used coefficient testing method (Chen et al., 2013; Jiang and Li, 2015), which is also a resampling method (Preacher and Hayes, 2008) and a good method for testing multiple mediation effects (Fritz et al., 2012; Hayes and Scharkow, 2013; Wen and Ye, 2014). ML is used for confirmatory factor analysis and structural equation modeling (Legg and Gray, 2000). This model can analyze to what extent different mediator variables positively or negatively affect the relationship between independent and dependent variables, that is, to analyze the significance and direction of the effects of different mediator variables.

Therefore, this article uses the “Bootstrap mediation” method and ML estimation method to analyze the mechanism of how different exercise durations affect the academic achievement of adolescents. Physical fitness and cognitive ability are used as mediator variables to analyze whether Physical fitness and cognitive ability have mediating effects on the academic achievement of adolescents under different exercise duration conditions. At the same time, the significance and direction of the direct and indirect effects produced by the two mediator variables are compared.

### 2.3. Variables and operational definitions

The dependent variable is academic achievement, mainly referring to students’ objective scores. The objective score is the exam score for subjects like Chinese, Math and English. Sun Zhijun et al. (Sun et al., 2009; Lei and Li, 2021) argued that since school papers vary, absolute scores cannot be compared, and standardized scores can ensure objectivity in quantifying scores. Therefore, the standardized score will be used as the indicator of the objective score. After standardization, the average standardized score for the three subjects is 70, and the standard deviation of the standardized score is 10.

The core independent variable is Physical activity duration. The CEPS survey investigated the time arrangement (hours) for physical activity participation on weekends among students. Based on the original answer options, if there was no Physical activity on weekends, it would be recorded as 0; if they exercised for one hour, it would be recorded as 1; if they exercised for two hours, it would be recorded as 2; if they exercised for three or more hours, it would be recorded as 3. The selection of weekend Physical activity time is based primarily on the following points: (1) The Education Department requires students to exercise for one hour every day. During school days, due to requirements such as sports classes, students may have ensured Physical activity time in school, resulting in small differences between student groups. However, students may lack exercise time on weekends, thus affecting their academic achievement. (2) Compared with previous studies on the relationship between academic achievement and Physical activity, some results showed that Physical activity had no correlation with academic achievement, possibly due to different effects on academic achievement from varying exercise durations. Therefore, this article chooses weekend Physical activity duration as the independent variable.

This study controlled for different variables based on the experience of scholars Asigbee et al. (2018) and Martin et al. (2018), as well as studies conducted by Wen (2015) and fully utilized the information contained in the survey data to control for various characteristics. Individual variables included age, gender, and ethnicity; family variables included household registration type, which primarily involved urban or rural household registration with agricultural households being 1 and urban households being 0; whether or not the participant was an only child, with 1 indicating being an only child and 0 indicating otherwise; parental education level, mainly defined according to the highest education level of either parent, with 0 indicating no education, 1 indicating elementary school, 2 indicating junior high school, 3 indicating high school, and 4 indicating university; family economic situation was divided into three categories: 0 for difficult, 1 for moderate, and 2 for wealthy; school characteristics included school location, which could be divided into main urban areas, secondary urban areas, urban–rural areas, townships, and rural areas depending on the school’s location; and grade, including data from seventh and ninth grades.

Mediating variables included cognitive ability and Physical fitness, where the CEPS questionnaire was used to test students’ logical thinking and problem-solving ability to reflect their cognitive level. Physical fitness was primarily assessed using height and weight data to calculate BMI scores, and based on the BMI formula and BMI classification standard, participants within the normal BMI range were assigned a score of 1, and others were assigned a score of 0 (Shen and Ma, 2022; Zhang et al., 2022). This was mainly because higher BMI scores are strongly associated with mortality (Xiang et al., 2022), and BMI scores can be used to evaluate student Physical fitness status and serve as a convenient method for measuring student Physical fitness (Han et al., 2022; Liu et al., 2023).

## 3. Results

### 3.1. Results of regression analysis of factors influencing adolescents’ academic achievement

According to Table 2, compared to students of other ethnicities, Han students had significantly lower academic achievement, especially after accounting for the mediating variable of cognitive ability. The education level of parents had a significant impact on students’ academic achievement, with higher parental education levels being associated with better academic achievement. School location also had a significant impact on academic achievement, with rural students outperforming urban students. Grade level was also a significant factor, with ninth-grade students performing better than seventh-grade students. Household registration type also had a significant effect on academic achievement, with agricultural household students performing better than non-agricultural household students. As for age, academic achievement significantly decreased as age increased. Family economic situation did not have a significant impact on academic achievement, but after adding the cognitive ability variable in Model 4, it was found that students from wealthy families had lower academic achievement than those from difficult families. Female students had significantly better academic achievement than male students. After adding Physical activity duration in Model 2, it was found that Physical activity duration had a significant impact on academic achievement, and different exercise durations had different effects on academic achievement. Adding the Physical fitness indicator in Model 3 showed that healthy students performed significantly better than unhealthy students. After adding academic achievement in Model 4, it was found that cognitive ability had a significant impact on academic achievement, with academic achievement improving with increasing cognitive ability. Adding both Physical fitness and cognitive ability variables in Models 3 and 4 resulted in large changes in the coefficient of the effect of Physical activity duration on academic achievement, especially after adding the cognitive ability variable, indicating that these two variables partially mediated the effect, leading to subsequent analysis using an intermediary model based on this model.

**Table 2:** Regression analysis of factors influencing adolescents’ academic achievement.

Variables (Compare)	Model I	Model 2	Model 3	Model 4
Han (Other)	−2.602^***^ (−3.68)	−2.654^***^ (−3.76)	−2.479^***^ (−3.49)	−4.320^***^ (−6.53)
Elementary school (none)	9.306^**^ (2.67)	9.078^**^ (2.61)	8.736^*^ (2.52)	6.517^*^ (2.02)
Junior high school (none)	11.200** (3.25)	10.940** (3.18)	10.440** (3.04)	7.066* (2.21)
High School (none)	13.280^***^ (3.84)	12.950^***^ (3.75)	12.440^***^ (3.61)	8.047^*^ (2.51)
University (none)	18.670^***^ (5.37)	18.250^***^ (5.25)	17.610^***^ (5.08)	11.280^***^ (3.49)
Border City (Main City)	2.765^***^ (4.32)	2.689^***^ (4.21)	2.815^***^ (4.38)	3.689^***^ (6.17)
Urban and Rural (Main City)	2.209^***^ (3.47)	2.214^***^ (3.49)	2.206^***^ (3.45)	4.513^***^ (7.57)
Township (main city)	3.240^***^ (5.52)	3.237^***^ (5.52)	3.215^***^ (5.45)	6.031^***^ (10.93)
Rural (main city)	3.251^***^ (5.40)	3.388^***^ (5.64)	3.599^***^ (5.94)	6.878^***^ (12.13)
Ninth Grade (VII)	6.171^***^ (9.31)	5.997^***^ (9.06)	5.866^***^ (8.80)	1.828^**^ (2.93)
Agricultural household (non-farm)	1.976^***^ (4.37)	1.929^***^ (4.28)	1.891^***^ (4.16)	1.822^***^ (4.30)
Age	−3.390^***^ (−12.41)	−3.338^***^ (−12.24)	−3.477^***^ (−12.65)	−1.405^***^ (−5.43)
Moderate (difficult)	0.677 (1.39)	0.588 (1.21)	0.545 (1.11)	−0.594 (−1.30)
Affluent (difficult)	−1.528 (−1.71)	−1.722 (−1.93)	−1.750 (−1.95)	−3.670^***^ (−4.38)
Non Only child (Yes)	0.016 (0.04)	0.035 (0.08)	−0.069 (−0.16)	1.458^***^ (3.55)
Female (Male)	12.570^***^ (33.14)	12.490^***^ (32.60)	12.30^***^ (31.84)	12.340^***^ (34.32)
Exercise for 1 h (no participation)		2.901^***^ (6.24)	2.848^***^ (6.08)	1.495^***^ (3.43)
Exercise for 2 h (no)		3.141^***^ (5.28)	3.067^***^ (5.12)	1.672^**^ (2.99)
Exercise for 3 h (no)		−1.879^**^ (−2.86)	−2.032^**^ (−3.07)	−1.791^**^ (−2.91)
Body shape (non-standard)			4.317^***^ (11.00)	3.013^***^ (8.23)
Cognitive ability				11.650^***^ (52.75)
_cons	236.3^***^ (44.56)	235.2^***^ (44.41)	235.1^***^ (44.27)	212.4^***^ (42.81)
*N*	18,337	18,337	18,009	18,009

Elementary school etc. is the education level of parents; t statistics in parentheses *p < 0.05, **p < 0.01, ***p < 0.001. Standard errors are indicated in parentheses.

The regression model results showed that Physical activity duration can directly affect students’ academic achievement and may also affect students’ academic achievement through mediating variables. To further verify whether Physical activity affects adolescent students’ academic achievement through mediating variables (Physical fitness and cognitive ability), this study used the “Bootstrap method” to analyze the mediating effect.

### 3.2. Differences between different durations of sports exercise

**Figure 1:**
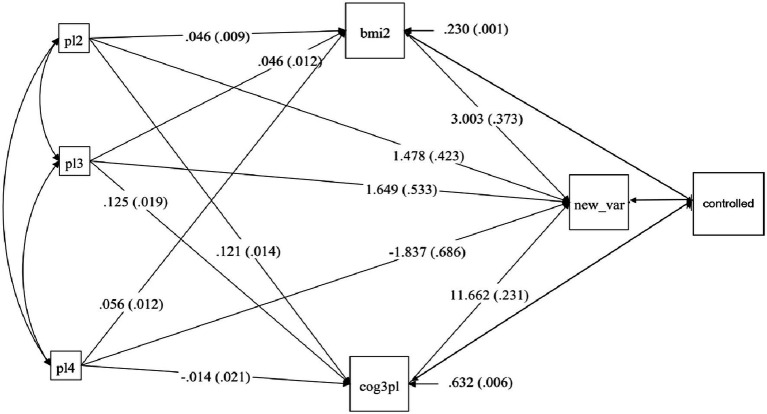
Path coefficients of the intermediary model. Model fit index RMSEA: 0.023; CFI: 0.987; TLI: 0.902; SRMR: 0.009; pl1 is 1 h of physical activity time, pl2 is 2 h of physical activity time, pl3 is 3 h of physical activity time; bmi2 = Physical fitness; cog3pl = cognitive ability; new_var = Academic achievement.

The effects of different Physical activity durations on adolescent students’ academic achievement vary. Compared to students who do not engage in Physical activity, students who exercise for one hour on weekends showed a significant improvement in academic achievement, with an increase of 3.031 standard deviations. Students who exercised for 2 hours on weekends also showed a significant improvement in academic achievement, with an increase of 3.249 standard deviations. However, when the exercise duration reached three or more hours, students’ academic achievement decreased by 1.837 standard deviations. Therefore, Physical activity duration has a significant impact on students’ academic achievement, and the effect varies depending on the duration of Physical activity. It can be observed that when the exercise duration is within 2 hours, students’ academic achievement improves as the duration of exercise increases. However, once this range is exceeded, academic achievement begins to decline (See Tables 3, 4; Figure 1).

**Table 3:** Bootstrap intermediary model path coefficients.

Variable	*b*	Ses	*t*	LLCl	ULCl
BMI2 ON					
PL2 (no)	0.046***	0.009	5.203	0.029	0.063
PL3 (no)	0.046***	0.012	3.961	0.024	0.068
PL4 (no)	0.056***	0.012	4.593	0.03	0.079
COG3PL ON					
PL2 (no)	0.121***	0.014	8.467	0.095	0.151
PL3 (no)	0.125***	0.019	6.596	0.089	0.164
PL4 (no)	−0.014	0.021	−0.646	−0.054	0.027
NEW_VAR ON					
PL2 (no)	1.478***	0.423	3.494	0.699	2.29
PL3 (no)	1.649**	0.533	3.097	0.485	2.64
PL4 (no)	−1.837**	0.686	−2.677	−3.131	−0.502
COG3PL	11.662***	0.231	50.581	11.19	12.087
Health (no)	3.003***	0.373	8.044	2.273	3.785

pl1 is 1 h of physical activity time, pl2 is 2 h of physical activity time, pl3 is 3 h of physical activity time, LLCI and ULCI = 95%. *p < 0.05, **p < 0.01, ***p < 0.001.

**Table 4:** Pathway analysis of the effect of physical fitness and cognitive ability on academic achievement.

Effect types	Effect	Boot SE	Boot LLCl	Boot ULCl	R1	R2
pl2 total effect	3.031*** (6.706)	0.452	2.2	3.924		
Direct effect	1.478*** (3.491)	0.423	0.699	2.29	48.76%	
total indirect effect	1.553*** (8.943)	0.174	1.239	1.915	51.24%	
pl2 → bmi2 → NEW_VAR	0.137*** (4.285)	0.032	0.083	0.208		8.82%
pl2 → COG3PL → NEW_VAR	1.416*** (8.338)	0.17	1.114	1.769		91.18%
pl3 total effect	3.249*** (5.601)	0.58	2.028	4.277		
Direct effect	1.649** (3.093)	0.533	0.485	2.64	50.75%	
total indirect effect	1.599*** (7.008)	0.228	1.171	2.048	49.22%	
pl3 → bmi2 → NEW_VAR	0.139*** (3.527)	0.039	0.071	0.22		8.69%
pl3 → COG3PL → NEW_VAR	1.461*** (6.537)	0.223	1.03	1.916		91.37%
pl4 total effect	−1.829* (−2.447)	0.748	−3.275	−0.404		
Direct effect	−1.837** (−2.677)	0.686	−3.131	−0.502	99.57%	
total indirect effect	0.008 (0.031)	0.254	−0.484	0.471	0.43%	
pl4 → bmi2 → NEW_VAR	0.168*** (3.905)	0.043	0.088	0.257		51.22%
pl4 → COG3PL → NEW_VAR	−0.16 (−0.645)	0.248	−0.62	0.313		48.78%

*t* statistics in parentheses ^*^p < 0.05, ^**^p < 0.01, ^***^p < 0.001. Standard errors are indicated in parentheses; R1: Ratio of indirect and direct to total effect; R2: Ratio of part indirect to total effect to total indirect.

### 3.3. Differences between intermediary paths

Compared to students who do not engage in Physical activity, different exercise durations have a significant effect on Physical fitness (*p* < 0.001). Participating in 1 h and 2 h of Physical activity can increase Physical fitness by 0.46 standard deviations, while a duration of three or more hours increases Physical fitness by 0.056 standard deviations. Results show that different exercise durations can improve students’ Physical fitness. The effect of different exercise durations on cognitive ability is also different. When the exercise duration is one or two hours, students’ cognitive ability significantly improves (*p* < 0.001), increasing by 0.121 and 0.125 standard deviations, respectively. When the exercise duration reaches three or more hours, cognitive ability decreases by 0.014 standard deviations, but the result is not statistically significant. Path results show that the effects of Physical fitness and cognitive ability on academic achievement are both significant (*p* < 0.001) and positive. Every one-unit increase in cognitive ability leads to an increase in academic achievement by 11.662 standard deviations, while Physical fitness can increase academic achievement by 3.003 standard deviations. These results indicate that Physical fitness and cognitive ability play a partial mediating role, and the mediating effect also changes with different exercise durations (See Table 3; Figure 1).

Different exercise durations have different effects on students’ Physical fitness and cognitive ability, and compared to students who do not engage in Physical activity, when the exercise duration reaches one hour, both cognitive ability and Physical fitness improve, leading to an improvement in academic achievement. The comparison of two mediating effects revealed that cognitive ability accounts for 91% of the total mediating effect, and although the proportion of Physical fitness’s mediating effect is relatively small, it still significantly improves students’ academic achievement.

When the exercise duration is two hours, academic achievement is improved by 1.461 standard deviations through the mediating effect of cognitive ability, indicating that cognitive ability has the best effect on academic achievement at this point. When the exercise duration reaches three or more hours, the mediating effect of cognitive ability changes, and cognitive ability does not continue to improve with increasing exercise duration. Instead, there is a slight but not statistically significant decrease in academic achievement by 0.16 standard deviations.

The effects of different exercise durations on students’ academic achievement through the mediating variables of Physical fitness are positive and significantly different (*p* < 0.001). Pathway results also show that as the exercise duration increases, the effect of Physical fitness on academic achievement continues to improve (See Table 4; Figure 1).

### 3.4. Analysis of heterogeneity among different genders

As shown in the table, different exercise durations have varying effects on aca-demic achievement for male and female students compared to those who do not participate in Physical activity. From the overall perspective, different exercise durations have a positive impact on the academic achievement of male students. The relationship between exercise duration and academic achievement presents an inverted U-shaped curve, with the best effect achieved at 2 h of exercise duration, resulting in academic achievement improvement of 6.29 standard deviations. In contrast to male students, female students achieve the best results when exercising for one hour, with an increase in academic achievement of 2.193 standard deviations. However, when the exercise duration reaches three hours, Physical activity significantly reduces female students’ academic achievement.

The direct effect results show that male students’ academic achievement increases with increasing exercise duration, as long as the exercise duration does not exceed two hours. However, when the exercise duration reaches three hours, Physical activity will reduce students’ academic achievement. Female students’ academic achievement im-proves when they exercise for an hour but significantly decreases with extended exercise durations. The indirect effect results show that exercise has a significant positive impact on male students’ academic achievement through cognitive abilities and Physical fitness levels. Female students mainly improve their cognitive abilities through Physical activity to enhance their academic achievement, and only one hour of exercise can promote improvement in Physical fitness and academic achievement (See Table 5).

**Table 5:** Heterogeneous path analysis.

Effect types	Boys			Girls		
	Effect	E.S.	Est./S.E.	Effect	E.S.	Est./S.E.
pl2 total effect	5.234***	0.721	7.257	2.193***	0.594	3.690
pl3 total effect	6.290***	0.830	7.578	1.294	0.803	1.612
pl4 total effect	0.334	0.913	0.365	−4.311***	1.223	−3.526
pl2 Direct effect	2.498***	0.664	3.762	0.512*	0.550	0.931
pl3 Direct effect	2.888***	0.794	3.636	0.063	0.736	0.085
pl4 Direct effect	−0.668	0.842	−0.794	−3.89**	1.120	−3.474
pl2 indirect effect	2.736***	0.307	8.903	1.681***	0.237	7.081
pl3 indirect effect	3.402***	0.348	9.785	1.231***	0.315	3.905
pl4 indirect effect	1.002**	0.359	2.790	−0.421	0.432	−0.975
pl2 → bmi2 → NEW_VAR	0.210***	0.056	3.726	0.074*	0.036	2.027
pl2 → COG3PL → NEW_VAR	2.526***	0.300	8.411	1.608***	0.232	6.942
pl3 → bmi2 → NEW_VAR	0.294***	0.073	4.033	−0.027	0.046	−0.599
pl23 → COG3PL → NEW_VAR	3.108***	0.343	9.070	1.259***	0.308	4.093
pl4 → bmi2 → NEW_VAR	0.264***	0.068	3.885	−0.015	0.065	−0.224
pl4 → COG3PL → NEW_VAR	0.738*	0.354	2.087	−0.406	0.423	−0.959

pl2 = 1 h of exercise, pl3 = 2 h of exercise, pl4 = 3 h of exercise; ^*^p < 0.05,^**^p < 0.01,^***^p < 0.001.

In summary, different exercise durations have different effects on students’ aca-demic achievement. When the exercise duration is within two hours, academic achievement improves as the exercise duration increases, and cognitive ability and Physical fitness play a partial mediating role. However, when the exercise duration reaches three or more hours, academic achievement decreases instead. At this point, the direct effect of Physical activity on academic achievement becomes negative, reducing students’ academic achievement. Although exercise duration can improve Physical fit-ness and cognitive ability, the mediating effect value is much lower than the direct effect value, so it cannot improve academic achievement but only mitigates the negative im-pact of exercise duration on academic achievement. Heterogeneity analysis results show that boys are more affected by exercise duration than girls, with boys having a higher improvement in academic achievement when exercising for two hours, while girls have a better academic achievement improvement when exercising for one hour.

### 3.5. Robustness test

Breen et al. (2013) proposed the KHB method, which not only decomposes the effects of continuous and categorical independent variables but also determines the existence of the mediating effect while measuring the contribution rate of the mediating effect. Therefore, this method is used here to conduct a robustness test on the previous content. The results show consistency with the previous content, that different exercise durations have varying effects on academic achievement, and the best exercise duration is 2 h. The results show passing the robustness test (Table 6).

**Table 6:** KHB mediated effect test of exercise duration on academic achievement: robustness test.

Independent variable	Total effect	Direct effect	Intermediate variables	Indirect effects	Indirect effects as a percentage
1 h	3.040***	1.524***	Physical Health	0.133*	4.37%
			Cognitive ability	1.384***	45.51%
2 h	3.275***	1.691**	Physical Health	0.137*	4.19%
			Cognitive ability	1.447***	44.19%
3 h	−1.821**	−1.813**	Physical Health	0.169**	−9.27%
			Cognitive ability	−0.178	9.75%

*t* statistics in parentheses *p < 0.05, **p < 0.01, ***p < 0.001.

## 4. Discussion

### 4.1. The direct impact of different durations of exercise on academic achievement

The study results indicate that different exercise durations have different effects on students’ academic achievement compared to students who do not engage in Physical activity. When the exercise duration is within two hours, it has the best effect on improving academic achievement. A certain amount of Physical activity can help students relax and improve studying efficiency. Scholars believe that Physical activity can affect academic achievement through various direct and indirect physiological, cognitive, emotional, and learning mechanisms (Fang, 2020). Extra-curricular physical activities can improve students’ classroom behavior and academic achievement (Álvarez-Bueno et al., 2017). However, when the exercise duration reaches three or more hours, Physical activity will hinder the improvement of students’ academic achievement. Studies have shown that performing 50 min of exercise every day improves academic achievement (Bai et al., 2020), while exercising for more than 75 min shows no improvement (Dwyer et al., 1983). Other studies have found that exercising for more than 120 min has a negative impact on academic achievement (Dong and Zhu, 2020). Therefore, although longer exercise may lead to better classroom performance and increased neural activity, excessive exercise time occupies too much of students’ study time, leading to a significant decrease in academic achievement when the exercise duration exceeds three hours.

In addition, the commonly used theory applied in research on the relationship between Physical activity and academic achievement is Activation Theory, which suggests an inverted-U relationship between task performance and activation level. Excessive activation levels may result in poor task performance (Wen, 2015). Therefore, appropriate Physical activity can improve adolescent students’ academic achievement.

### 4.2. Effect of physical fitness and cognitive ability on academic achievement

This study found that Physical fitness has a significant impact on students’ academic achievement. Different exercise durations can improve students’ physical fitness. Compared with non-healthy students who are either overweight or underweight, students who maintain a normal body shape have better academic achievement. However, the relationship between adolescent Physical fitness and academic achievement is complex. Some scholars believe that Physical fitness is the key indicator that affects academic achievement (Liang and Zhang, 2016; Asigbee et al., 2018). Although this study found that Physical fitness can affect students’ academic achievement, factors that truly affect academic achievement may not be Physical fitness. Individuals with obesity or underweight features are more likely to face discrimination, bullying, even stigmatization, and they struggle to obtain the same resources as those with normal body shape (Lumeng et al., 2010). And other factors such as obesity may also lead to a decline in self-esteem and confidence, causing anxiety and eventually resulting in a decrease in academic achievement (Kristjánsson et al., 2010). Physical activity can improve body shape, Physical fitness, and enhance student’s self-esteem (Biddle et al., 2019), thereby improving their academic achievement.

Compared to students who do not participate in Physical activity, participating in Physical activity can improve students’ cognitive abilities and have an impact on their academic achievement. The research results indicate that moderate Physical activity can significantly improve students’ cognitive abilities. Physical activity is believed to immediately increase the physiological arousal level of young people, thereby promoting cognitive performance by increasing attention allocation (Roig et al., 2013). Cotman and Berchtold (2002) suggest that Physical activity can trigger a series of neurobiological mechanisms that may enhance memory processing in humans. The primary physiological mechanism of Physical activity functioning on cognitive ability is that it can affect cellular processes and improve energy utilization efficiency (Bélanger et al., 2011). Based on energy utilization, cells can efficiently carry out various biochemical reactions and synthesize substances needed to maintain the survival of neurons, such as neurotransmitters (Dishman et al., 2006; Thomas et al., 2012; Roig et al., 2013). Therefore, Physical activity can promote the development of cognitive ability (XIA, Xia et al., 2018), thereby improving academic achievement. However, some studies have shown that excessive Physical activity can negatively impact cognitive ability due to the body and psychological burden (Tomporowski et al., 2008).

### 4.3. Differences in physical activity between genders

Through heterogeneity comparison, it was found that the effect of Physical activity on academic achievement differs between boys and girls. Boys exhibit a greater positive effect of Physical activity on academic achievement, with the largest improvement achieved at a 2-h exercise duration. Girls show a significant improvement in academic achievement after exercising for 1 h, but their academic achievement decreases with further increase in exercise duration. Data analysis also shows that girls’ overall academic achievement is better than boys by 12.61 standard deviations, and the marginal contribution of Physical activity to academic achievement for girls faces a decreasing trend. In contrast, boys are more affected by marginal effects, so exercising over the weekend can improve their academic achievement, which is consistent with the findings of Fang (2020). Analysis on the mediating variable of Physical fitness shows that boys significantly increase their Physical fitness level through Physical activity, while girls’ Physical fitness level only improves significantly when exercising for 1 h, which may be due to differences in the intensity of Physical activity between boys and girls. Research shows that most women choose public sports venues and parks (Meng et al., 2005), and walking as their preferred form of exercise (Liu et al., 2013; Wang et al., 2015), which are relatively low-intensity exercise activities with less energy consumption, therefore, their impact on Physical fitness is not significant. Boys’ cognitive abilities are improved by Physical activity of different durations, while girls show a higher improvement in cognitive abilities after 1 h of Physical activity, which may be related to the content of physical activities chosen by girls such as walking forms of exercise, which may not enhance cognitive ability and may even affect their use of study time.

## 5. Conclusion

1. Different durations of physical activity have different effects on academic performance, and there is an optimal activity time for students’ academic performance. When the exercise duration is two hours, the improvement in students’ academic performance is the greatest.
2. Physical activity can improve students’ physical fitness, enhance their cognitive ability, and thus improve their academic performance.
3. Different durations of physical activity can improve students’ physical fitness, but there is an optimal value for the improvement of cognitive ability with respect to the duration of physical activity. There are also differences between male and female students. For the improvement of cognitive ability, the optimal activity time for male students is two hours, while for female students, it is one hour.

## 6. Recommendations

1. Parents and schools should attach great importance to the value of Physical activity, encourage students to participate in Physical activity, and promote academic growth by improving cognitive abilities and Physical fitness.
2. Students should reasonably arrange the duration of Physical activity, keeping it around 2 h to more effectively promote academic achievement.
3. Schools can assign physical homework on weekends based on students’ physical foundation and interests, set the required completion time to be around 2 h, which can improve Physical fitness as well as academic achievement.

## Data availability statement

The original contributions presented in the study are included in the article/supplementary material, further inquiries can be directed to the corresponding author.

## Ethics statement

Ethical review and approval was not required for the study on human participants in accordance with the local legislation and institutional requirements. Written informed consent from the patients/participants or patients/participants' legal guardian/next of kin was not required to participate in this study in accordance with the national legislation and the institutional requirements.

## Author contributions

GL and XL: conceptualization. GL, XL, and WL: methodology and data curation. GL: writing—original draft. All authors have read and agreed to the published version of the manuscript.

## Conflict of interest

The authors declare that the research was conducted in the absence of any commercial or financial relationships that could be construed as a potential conflict of interest.

## Publisher’s note

All claims expressed in this article are solely those of the authors and do not necessarily represent those of their affiliated organizations, or those of the publisher, the editors and the reviewers. Any product that may be evaluated in this article, or claim that may be made by its manufacturer, is not guaranteed or endorsed by the publisher.

## References

[ref1] Álvarez-Bueno C. Pesce C. Cavero-Redondo I. Sánchez-López M. Garrido-Miguel M. Martínez-Vizcaíno V. (2017). Academic achievement and physical activity: a meta-analysis. Pediatrics 140:1498. doi: 10.1542/peds.2017-1498, PMID: 29175972

[ref2] Asigbee F. M. Whitney S. D. Peterson C. E. (2018). The link between nutrition and physical activity in increasing academic achievement. J. Sch. Health 88, 407–415. doi: 10.1111/josh.12625, PMID: 29748999

[ref3] Bai S. Pan Z. Teng H. (2020). An empirical study of the impact of exercise on academic performance in Middle school students. China Sport Sci. 40, 64–72.

[ref4] Bangsbo J. Krustrup P. Duda J. Hillman C. Andersen L. B. Weiss M.. (2016). The Copenhagen consensus conference 2016: children, youth, and physical activity in schools and during leisure time. Br. J. Sports Med. 50, 1177–1178. doi: 10.1136/bjsports-2016-096325, PMID: 27354718 PMC5036221

[ref5] Bélanger M. Allaman I. Magistretti P. J. (2011). Brain energy metabolism: focus on astrocyte-neuron metabolic cooperation. Cell Metab. 14, 724–738. doi: 10.1016/j.cmet.2011.08.016, PMID: 22152301

[ref6] Biddle S. J. H. Ciaccioni S. Thomas G. Vergeer I. (2019). Physical activity and mental health in children and adolescents: an updated review of reviews and an analysis of causality. Psychol. Sport Exerc. 42, 146–155. doi: 10.1016/j.psychsport.2018.08.011

[ref7] Breen R. Karlson K. B. Holm A. (2013). Total, direct, and indirect effects in Logit and Probit models. Sociol. Methods Res. 42, 164–191. doi: 10.1177/0049124113494572

[ref8] Carlson S. A. Fulton J. E. Lee S. M. Maynard L. M. Brown D. R. Kohl H. W. III. (2008). Physical education and academic achievement in elementary school: data from the early childhood longitudinal study. Am. J. Public Health 98, 721–727. doi: 10.2105/AJPH.2007.117176, PMID: 18309127 PMC2377002

[ref9] Chan J. K.-w. Hui S. S.-c. (2016). Physical activity participation was not associated with academic performance in ethnic minority students: 3756 board #195 June 4, 8: 00 AM -9: 30 AM. Med. Sci. Sports Exerc. 48:1049. doi: 10.1249/01.mss.0000488159.68286.c8

[ref10] Chen R. Zheng Y. H. Liu W. J. (2013). Mediation analysis: principles, procedures, bootstrap methods and applications. J. Market. Sci. 9, 120–135.

[ref11] Coe D. P. Pivarnik J. M. Womack C. J. Reeves M. J. Malina R. M. (2006). Effect of physical education and activity levels on academic achievement in children. Med. Sci. Sports Exerc. 38, 1515–1519. doi: 10.1249/01.mss.0000227537.13175.1b 16888468

[ref12] Cotman C. W. Berchtold N. C. (2002). Exercise: a behavioral intervention to enhance brain health and plasticity. Trends Neurosci. 25, 295–301. doi: 10.1016/S0166-2236(02)02143-4, PMID: 12086747

[ref13] Dishman R. K. Berthoud H.-R. Booth F. W. Cotman C. W. Edgerton V. R. Fleshner M. R.. (2006). Neurobiology of exercise. Obesity 14, 345–356. doi: 10.1038/oby.2006.46 16648603

[ref14] Dong Y. M. Zhu C. G. (2020). A study on the influence of extra-curricular sports on academic performance of teenagers: on the mediating effect of non-cognitive ability. J. Sports Res. 34, 52–62.

[ref16] Dwyer T. Coonan W. Leitch D. Hetzel B. Baghurst R. (1983). An investigation of the effects of daily physical activity on the health of primary school students in South Australia. Int. J. Epidemiol. 12, 308–313. doi: 10.1093/ije/12.3.308, PMID: 6629620

[ref17] Fang L. (2020). The effect of physical exercise on adolescents’ cognitive ability and academic performance. China Sport Sci. 40, 35–41.

[ref18] Fritz M. S. Taylor A. B. Mackinnon D. P. (2012). Explanation of two anomalous results in statistical mediation analysis. Multivar. Behav. Res. 47, 61–87. doi: 10.1080/00273171.2012.640596 PMC3773882 24049213

[ref19] Guthold R. Stevens G. A. Riley L. M. Bull F. C. (2020). Global trends in insufficient physical activity among adolescents: a pooled analysis of 298 population-based surveys with 1·6 million participants. Lancet Child Adolesc Health 4, 23–35. doi: 10.1016/S2352-4642(19)30323-2, PMID: 31761562 PMC6919336

[ref20] Han Z. Lun Y. Liu T. (2022). Influence of URBAN facilities and environment on junior MIDDLE school STUDENTS' physical health: a case study of Dalian city. Hum. Geogr. 37, 99–109. (in Chinese)

[ref21] Hao W. Yi J. (2022). Dilemma and countermeasures of delayed after-school sports Service in Primary and Secondary Schools under the policy of double reduction. Sports Cult. Guide 10, 95–101. (in Chinese)

[ref22] Hayes A. F. Scharkow M. (2013). The relative trustworthiness of inferential tests of the indirect effect in statistical mediation analysis: does method really matter? Psychol. Sci. 24, 1918–1927. doi: 10.1177/0956797613480187, PMID: 23955356

[ref23] Hillman C. Erickson K. Kramer A. (2008). Be smart, physical activity your heart: physical activity effects on brain and cognition. Nat. Rev. Neurosci. 9, 58–65. doi: 10.1038/nrn2298, PMID: 18094706

[ref24] Jiang C. M. Li S. (2015). Mediation analysis and the application of bootstrap in mediation analysis. Psychol. Explor. 35, 458–463.

[ref25] Kristjánsson Á. L. Sigfúsdóttir I. D. Allegrante J. P. (2010). Health behavior and academic achievement among adolescents: the relative contribution of dietary habits, physical activity, body mass index, and self-esteem. Health Educ. Behav. 37, 51–64. doi: 10.1177/1090198107313481, PMID: 18541647

[ref26] Legg J. A. Gray D. A. (2000). Performance bounds for polynomial phase parameter estimation with nonuniform and random sampling schemes. IEEE Trans. Signal Process. 48, 331–337. doi: 10.1109/78.823961

[ref27] Lei W. Li Z. (2021). The effect of the non-cognitive ability on Middle school students’ academic achievement: an empirical analysis based on CEPS. J. Central China Normal Univ. 60, 154–163. (in Chinese)

[ref28] Liang Z. Zhang Y. (2016). Empirical research of physical shape, cardiopulmonary function and academic scores based on the empirical research of physical shape, cardiopulmonary function and academic scores based on the comparison between National Guideline of student physical fitness 2014 and 2007. Sports Sci. 37, 89–97. (in Chinese)

[ref29] Liu J. Sun H. Beets M. W. Probst J. C. (2013). Assessing natural groupings of common leisure-time physical activities and its correlates among US adolescents. J. Phys. Act. Health 10, 470–479. doi: 10.1123/jpah.10.4.470, PMID: 22820608

[ref30] Liu D. Shen L. Yu B. Yu W. Jia P. Yang S. (2023). Influence of social capital changes on body mass index of Chinese students during COVID-19 lockdown. Modern Prevent. Med. 50, 317–322. (in Chinese)

[ref31] Lumeng J. C. Forrest P. Appugliese D. P. Kaciroti N. Corwyn R. F. Bradley R. H. (2010). Weight status as a predictor of being bullied in third through sixth grades. Pediatrics 125, e1301–e1307. 20439599 10.1542/peds.2009-0774 PMC4174570

[ref32] Martin A. Booth J. N. Laird Y. Sproule J. Reilly J. J. Saunders D. H.. (2018). Physical activity, diet and other behavioural interventions for improving cognition and school achievement in children and adolescents with obesity or overweight. Cochrane Database Syst. Rev. 2018, 3:CD009728. doi: 10.1002/14651858.CD009728.pub4 PMC6491168 29376563

[ref33] Masoomi H. Taheri M. Irandoust K. H’Mida C. Chtourou H. (2020). The relationship of breakfast and snack foods with cognitive and academic performance and physical activity levels of adolescent students. Biol. Rhythm. Res. 51, 481–488. doi: 10.1080/09291016.2019.1566994

[ref34] Maynard E. Coonan W. Worsley A. Dwyer T. Baghurst P. (1987). The development of the lifestyle education program in Australia. Cardiovascular Risk Factors in Children: Epidemiology and Prevention 123–149.

[ref35] McPherson A. Mackay L. Kunkel J. Duncan S. (2018). Physical activity, cognition and academic performance: an analysis of mediating and confounding relationships in primary school children. BMC Public Health 18:936. doi: 10.1186/s12889-018-5863-1, PMID: 30064394 PMC6069778

[ref36] Meng W. Guo Y. Zou X. Guo-Ying Q. (2005). Investigation and research on Chinese Women’s participation in mass sports and its general condition in the current period. J. Beijing Sport Univ. 28, 295–298.

[ref37] News X. (2021). 2019 national student physical health attainment excellent rate of 23.8%. Available at: https://baijiahao.baidu.com/s?id=1709863679300048449 (Accessed April 08, 2023).

[ref38] Pei D. Gao P. (2013). Studying on the Physical & Mental Health and the sport participation behaviors of migrant Workers’ Children. Sports Sci. 34, 94–98. (in Chinese)

[ref39] Preacher K. J. Hayes A. F. (2008). Asymptotic and resampling strategies for assessing and comparing indirect effects in multiple mediator models. Behav. Res. Methods 40, 879–891. doi: 10.3758/BRM.40.3.879, PMID: 18697684

[ref15] Reed A. N. (2016). Physical activity, fitness, cognitive function, and academic achievement in children: A systematic review. Med. Sci. Sports Exerc. 48, 1197–222. 27182986 10.1249/MSS.0000000000000901 PMC4874515

[ref40] Roig M. Nordbrandt S. Geertsen S. S. Nielsen J. B. (2013). The effects of cardiovascular exercise on human memory: a review with meta-analysis. Neurosci. Biobehav. Rev. 37, 1645–1666. doi: 10.1016/j.neubiorev.2013.06.012, PMID: 23806438

[ref41] Shen K. Ma W. (2022). Research on the characteristics and influence of adolescents on the aesthetic orientation of physical education curriculum. China Sport Sci. 42, 50–59. (in Chinese)

[ref42] Singh A. S. Saliasi E. van den Berg V. Uijtdewilligen L. de Groot R. H. M. Jolles J.. (2019). Effects of physical activity interventions on cognitive and academic performance in children and adolescents: a novel combination of a systematic review and recommendations from an expert panel. Br. J. Sports Med. 53, 640–647. doi: 10.1136/bjsports-2017-098136, PMID: 30061304

[ref001] Sun Z. Liu Z. Sun B. (2009). Children’s achievements and their families and schools: a study based on the rural areas in Gansu province. Journal of Beijing Normal University (Social Sciences) 5, 103–115.

[ref43] Thomas A. G. Dennis A. Bandettini P. A. Johansen-Berg H. (2012). The effects of aerobic activity on brain structure. Front. Psychol. 3:86. doi: 10.3389/fpsyg.2012.00086 22470361 PMC3311131

[ref44] Tomporowski P. D. Davis C. L. Miller P. H. Naglieri J. A. (2008). Exercise and children’s intelligence, cognition, and academic achievement. Educ. Psychol. Rev. 20, 111–131. doi: 10.1007/s10648-007-9057-0, PMID: 19777141 PMC2748863

[ref45] Tomporowski P. D. Pesce C. (2019). Exercise, sports, and performance arts benefit cognition via a common process. Psychol. Bull. 145, 929–951. doi: 10.1037/bul0000200, PMID: 31192623

[ref46] Tremblay M. S. Inman J. W. Douglas Willms J. (2000). The relationship between physical activity, self-esteem, and academic achievement in 12-year-old children. Pediatr. Exerc. Sci. 12, 312–323. doi: 10.1123/pes.12.3.312

[ref47] Visier-Alfonso M. E. Álvarez-Bueno C. Sánchez-López M. Cavero-Redondo I. Martínez-Hortelano J. A. Nieto-López M.. (2021). Fitness and executive function as mediators between physical activity and academic achievement: mediators between physical activity and academic achievement. J. Sports Sci. 39, 1576–1584. doi: 10.1080/02640414.2021.1886665, PMID: 33612080

[ref48] Wang F. Jiang C. Wang M. Zhang Y. (2015). Study on the characteristics and F actors of intergenerational change in Chinese Female's physical exercise. China Sport Science 35, 24–34. (in Chinese)

[ref49] Wen X. (2015). Influence of physical exercise on cognitive function and academic performance in children: history, current status and future. China Sport Sci. 35, 73–82.

[ref50] Wen Z. Ye B. (2014). Analyses of mediating effects: the development of methods and models. Adv. Psychol. Sci. 22:731. doi: 10.3724/SP.J.1042.2014.00731

[ref51] World Health Organization. (2022). Physical activity, Vol. 2023.4.17.

[ref52] Wunsch K. Kasten N. Fuchs R. (2017). The effect of physical activity on sleep quality, well-being, and affect in academic stress periods. Nature Sci. Sleep 9, 117–126. doi: 10.2147/NSS.S132078, PMID: 28490911 PMC5414656

[ref53] Xia H.. (2018). The brain mechanisms of the physical exercise enhancing cognitive function. Adv. Psychol. Sci. 26, 1857–1868. doi: 10.3724/SP.J.1042.2018.01857

[ref54] Xiang H. Yang R. Tu J. Guan X. Tao X. (2022). Health impacts of high BMI in China: terrible present and future. Int. J. Environ. Res. Public Health 19:173. doi: 10.3390/ijerph192316173, PMID: 36498245 PMC9739093

[ref55] Yu C. C. W. Chan S. Cheng F. Sung R. Y. T. Hau K. T. (2006). Are physical activity and academic performance compatible? Academic achievement, conduct, physical activity and self-esteem of Hong Kong Chinese primary school children. Educ. Stud. 32, 331–341. doi: 10.1080/03055690600850016

[ref56] Zhang Y. Su F. Song Y. Lu J. (2022). Associations between physical fitness index and body mass index in Tibetan children and adolescents in different high-altitude areas: based on a study in Tibet, China. Int. J. Environ. Res. Public Health 19:155. doi: 10.3390/ijerph191610155 PMC9408390 36011789

